# Comparison of the Prognostic Value of Three Different Single HLA Based Antibody Detection Assays

**DOI:** 10.1111/tan.70734

**Published:** 2026-04-25

**Authors:** Falko M. Heinemann, Michael Hallensleben, Karina Althaus, Klemens Budde, Gunilla Einecke, Ute Eisenberger, Andrea Ender, Thorsten Feldkamp, Florian Grahammer, Martina Guthoff, Christopher Holzmann‐Littig, Christian Hugo, Teresa Kauke, Stephan Kemmner, Martina Koch, Nils Lachmann, Monika Lindemann, Christian Morath, Martin Nitschke, Lutz Renders, Sabine Scherer, Gerald Schlaf, Julian Stumpf, Vedat Schwenger, Florian Sommer, Bernd M. Spriewald, Caner Süsal, Murielle Verboom, Daniel Zecher, Malte Ziemann

**Affiliations:** ^1^ University Hospital Essen, Institute for Transfusion Medicine University Duisburg‐Essen Essen Germany; ^2^ Hannover Medical School Institute of Transfusion Medicine and Transplant Engineering Hannover Germany; ^3^ University Hospital of Tuebingen Institute for Clinical and Experimental Transfusion Medicine Tübingen Germany; ^4^ Charité‐Universitätsmedizin Berlin, Medizinische Klinik m. S. Nephrologie Berlin Germany; ^5^ Universitätsmedizin Göttingen, Klinik für Nephrologie und Rheumatologie Göttingen Germany; ^6^ Knappschaftskrankenhaus Bottrop, Klinik für Innere Medizin II – Nephrologie und Rheumatologie Bottrop Germany; ^7^ Klinikum der Landeshauptstadt Stuttgart, Institute for Transfusion Medicine Stuttgart Germany; ^8^ University Hospital of Schleswig‐Holstein, Transplantationszentrum Lübeck Germany; ^9^ University Hospital Hamburg Eppendorf, III. Medizinische Klinik Und Poliklinik für Nephrologie, Rheumatologie und Endokrinologie Hamburg Germany; ^10^ University Hospital of Tuebingen, Medizinische Klinik IV Sektion Nieren‐ und Hochdruckkrankheiten Tübingen Germany; ^11^ Klinikum Rechts der Isar der Technischen Universität München, Nephrologie München Germany; ^12^ University Hospital Carl Gustav Carus, Medizinische Klinik III Dresden Germany; ^13^ Klinikum der Universität München, Labor für Immungenetik München Germany; ^14^ Klinikum der Universität München, Transplantationszentrum München Germany; ^15^ Klinik für Allgemein‐, Viszeral‐ und Transplantationschirurgie, Universitätsmedizin Mainz Germany; ^16^ Charité‐Universitätsmedizin Berlin, Institute for Transfusion Medicine, HLA‐Labor Berlin Germany; ^17^ University Hospital Heidelberg, Zentrum für Innere Medizin, Nephrologie Heidelberg Germany; ^18^ Department of Nephrology and Hypertension Klinikum Nuremberg, and Paracelsus Medical University Nuremberg Germany; ^19^ University Hospital Heidelberg, Institut für Immunologie, Transplantationsimmunologie Heidelberg Germany; ^20^ University Hospital Halle, Institute for Transfusion Medicine Halle Germany; ^21^ Klinikum der Landeshauptstadt Stuttgart, Klinik für Nieren‐, Hochdruck‐ und Autoimmunerkrankungen Stuttgart Germany; ^22^ University Hospital Augsburg, Klinik für Allgemein‐, Viszeral‐ und Transplantationschirurgie Augsburg Germany; ^23^ University Hospital Erlangen, Medizinische Klinik 5 – Hämatologie und Internistische Onkologie Erlangen Germany; ^24^ Koç University School of Medicine, Transplant Immunology Research Center of Excellence, TIREX Istanbul Turkey; ^25^ Department of Nephrology University Hospital Regensburg Regensburg Germany; ^26^ University Hospital of Schleswig‐Holstein, Institute for Transfusion Medicine Lübeck Germany

**Keywords:** donor‐specific HLA antibodies, graft survival, kidney transplantation, Luminex, single antigen beads

## Abstract

Single antigen (SA) tests are indispensable for an accurate HLA antibody identification in sera of transplant patients. Three commercial tests using purified single antigens combined with microbead or microarray technology are currently available. This study aims to determine the prognostic value of these tests prior to kidney transplantation. Forty‐nine pretransplant sera with donor‐specific antibodies (DSA) from patients who underwent kidney transplantation from a living donor were selected from a previous multicentre study. All sera were tested by a new microspot SA test from BAG and bead array SA assays from Immucor/Werfen (IMM) and One Lambda/ThermoFisher (OLI). AMR‐free survival within 6 months (AMR‐S) and 10‐year death‐censored graft survival (10yGS) were compared to DSA‐negative patients from the original study and evaluated according to (1) number of SA tests classifying the serum as DSA‐positive (DSA+), and (2) number of SA tests detecting at least one identical DSA specificity. In part (1), the 22 patients classified as DSA‐positive by all tests had lowest AMR‐S and 10yGS. OLI was most sensitive and classified all sera as DSA‐positive that were DSA‐positive by BAG and/or IMM. However, the 14 patients who were DSA‐negative by both BAG and IMM, had similar AMR‐S and 10yGS like patients without any DSA. Overall, BAG and IMM had comparable sensitivities. In part (2), at least one identical DSA was detected by all tests in 18 patients, who had worse AMR‐S and 10yGS. In 14 patients, the same DSA was detected by IMM and OLI, or by BAG and OLI, respectively. These patients had lower AMR‐S, but no significant difference in 10yGS. In 17 sera, DSA were detectable by one or more tests, but no specificity was positive in more than one assay (in all sera DSA were detected by OLI, in one additionally another DSA by IMM and in two another DSA by BAG). AMR‐S and 10yGS were similar to DSA‐negative patients. Overall, all three SA assays were suitable for the reliable detection of strong DSA. OLI was shown to be the most sensitive assay, but also prone to possible false positive results defined by the lack of an association with impaired outcomes. Future studies are needed to determine how many OLI‐only reactions are caused by very weak DSA and how many by reactions with denatured beads. While such reactions were rare for BAG and IMM assays, these tests missed some DSA associated with an increased risk for AMR.

**Abbreviations**
AMR: antibody mediated rejection
ATG: anti thymocyte globulin
DSA: donor specific HLA antibodies
GS: death censored graft survival
MFI: mean fluorescence intensity
PRA: panel reactive antibodies
SA: single antigen

## 1 Introduction

The risk for antibody‐mediated rejections (AMR) and graft loss after kidney transplantation is increased for patients with antibodies against any of the donor's HLA (HLA DSA) when detected prior to transplantation [1, 2]. There is still considerable debate about the optimal parameters for risk stratification within patients with preformed DSA, but a general benefit of Single Antigen (SA) tests for an accurate HLA antibody identification is highly recognised in the kidney transplantation immunology field [3, 4, 5]. So, the objective of this work is to compare the prognostic value of all three available SA assays prior to kidney transplantation.

Today, many DSA characteristics can be determined in vitro (like the IgG subtype, the ability to bind complement, the mean fluorescence intensity (MFI) in SA testing). Technically, most laboratories rely on the well‐established and frequently used SA testing assays by One Lambda/Thermo Fisher (OLI) and Immucor/Werfen (IMM) based on the microbead technology in combination with Luminex flow analysers [6, 7]. Although Luminex based screening and specification assays have different sensitivities for the definition of anti HLA antibodies, most laboratories use a step wise screening strategy starting with a screening for HLA class I and II positivity before proceeding to expensive SA specification assays. However, this procedure could be prone to miss some relevant low reactive antibodies [8], why one could consider to perform direct SA bead testing without a preceded Luminex screening. In addition, there are other limitations of the Luminex SA bead based methodology: The MFI positivity threshold, which is a critical parameter in SA bead analysis, is still a subject of ongoing discussion. One key factor to be considered is the semiquantitative MFI measurement applied when the Luminex instrument is used [9]. Without harmonisation, the level of antibodies cannot be estimated accurately, leading to non‐comparable results between different laboratories. In a contemporary approach, the MFI values serve more as an orientation tool and the comparison with reaction patterns of historic sera, diluted sera and the assessment of serial variations in anti‐HLA reactions became more relevant for the clinical interpretation [10, 11, 12].

Recently, a new multiplex immunodiagnostic HISTO SPOT microarray based on the automated MR.SPOT platform provided by the company BAG Diagnostics was released, which also enables HLA antibody specification with purified single HLA antigens [13, 14]. This new assay was shown to be able to predict a cross‐match result and the relative sensitivity compared with the established Luminex bead based assays was evaluated [15]. So, there are now three commercial tests using purified single antigens available combined with microbead or microarray technology, but little is known about their comparative prognostic value for the clinical course of the patients.

Our study patients originate from a previous study stratifying the risk in patients with DSA before living donor kidney transplantation by different crossmatch methods [16]. The goal of the current study was to evaluate the prognostic utility of all three available SA tests using AMR‐free survival and 10‐year‐graft survival as outcome parameters. To achieve this, we re‐analysed DSA positive pretransplant sera from patients who underwent living donor kidney transplantations.

## 2 Materials and Methods

### 2.1 Study Cohort

In brief, this study originates from a multicentre cohort consisting of kidney transplant patients transplanted during the years 2012 until 2015 and established to examine the prognostic value of preformed DSA. It comprises 49 DSA‐positive cases and 1216 DSA‐negative controls.

Most cases were recruited from a study examining a total of 4233 consecutive adult renal transplant patients from 18 German transplant centres, of whom 4132 had complete follow‐up data [1]. Of these patients, 1324 had received a living donor transplant. 1216 of those had no DSA prior to transplant, while 108 had received a kidney from a living donor despite known preformed DSA. Additional seven patients with preformed DSA from two other German transplant centres were recruited for a follow‐up study comparing different crossmatch methods [16]. For only 49 out of those 115 DSA‐positive patients sufficient aliquots of pretransplant sera were available for the current study. All 1216 living donor transplant recipients from the original study without any pretransplant DSA were used as controls. This number differs slightly from the number of controls reported in the original publication, because only DSA against HLA‐A, ‐B, ‐C, ‐DRB1 and DQB1 antigens had been considered in that initial study [1].

The original antibody testing had been performed by OLI or IMM SA tests, as the microarray test from BAG was unavailable in those years. Only complement‐dependent cytotoxicity crossmatches had been performed prior to transplantation. Clinical outcome data were taken from the previous studies [1]. For every patient, only a single serum sample stored prior to transplantation was proceeded to the HLA antibody diagnostics of the current study using the three SA assays.

HLA‐class I and II typing of patients and living donors was performed at first field resolution level for the HLA‐A, ‐B, ‐C, ‐DR and ‐DQ loci enabling at least the definition of serological equivalents. In addition, second field typing and/or typing of additional loci was performed, if needed to determine whether preformed HLA antibodies were donor specific. Two patient‐donor pairs were excluded from the cohort when no DNA was available any more for further typing.

Antibody mediated rejection (AMR) free survival within 6 months post transplantation and 10‐year death‐censored graft survival (10yGS) were evaluated according to (1) number of SA tests classifying the serum as ‘DSA‐positive’ (number of SA tests detecting at least one DSA in the serum), and (2) number of SA tests detecting at least one identical DSA specificity. A clarifying overview about the distinction of the study groups is available as Supporting Information to this work (Figure S1). The clinical and research activities being reported are consistent with the principles of the declaration of Istanbul as outlined in the ‘Declaration of Istanbul on Organ Trafficking and Transplant Tourism’. The study was approved by the ethics committee of the University of Lübeck.

### 2.2 HLA Antibody Diagnostics

In brief, all 49 sera with sufficient aliquots were tested by a microspot array SA test from BAG (HISTO SPOT HLA AB ID Class I and II, BAG Diagnostics GmbH, Germany, in the following referred to as ‘BAG’) and two SA microbead arrays from Immucor (LifeScreen LSA Class I + II, Lifecodes/Immucor Werfen Inc., Stamford, CT, USA, in the following referred to as ‘IMM’) and from One Lambda (LABScreen SAB class I + II, One Lambda/Thermo Fisher, Canoga Park, CA, USA, in the following referred to as ‘OLI’). All three assays were performed following the manufacturer's instructions and all patient sera were analysed after at least two freeze–thaw cycles. The BAG and OLI assays were performed in the same lab, whereas the IMM testing was performed in another lab. All three SA tests were evaluated independently for positivity.

The HISTO SPOT microspot test system applied a multiplex immunodiagnostic microarray on the automated MR.SPOT platform provided by the same company [13, 14, 15]. The array consisted of 98 recombinant single HLA class I antigens and 126 recombinant HLA class II antigens, representing an antigen panel composition comparable to the IMM and OLI assays. Each antigen was printed on the bottom of the well to have an independent spot available for analysis. The resulting antibody signals (coloured dots in the bottom of each test well) were photographed by the MR.SPOT processor and the image was transferred into the HISTO MATCH interpretation software. For each serum, the image analysis software determined the colour intensity of the background and of each spot in the array. Based on the background and the variability of the specific spot during the quality control tests performed by the manufacturer before release of the lot, the software calculated an individual cut off value for the colour intensity of each spot. Spots with reactions above this cut off value were considered as positive. There was no additional serum treatment done for the BAG assay before setup of the test.

In addition, the well‐known SA microbead assays by One Lambda/Thermo Fisher and Immucor/Werfen were performed with the same stored pretransplant sera as established for routine purposes. For OLI the sera were treated with EDTA prior to testing, whereas the sera were heat inactivated and filtrated prior to the setup of the IMM assay. The OLI threshold for the definition of positivity was a normalised MFI of at least 500. For IMM, a positivity cut‐off greater or equal to 2 was applied according to the recommendation of the manufacturer. More specifically: A bead was considered positive, if two or more out of three scores calculated by the analysis software were positive (at least 1500 background corrected MFI, background corrected ratio of 3 or higher and antigen density adjusted background corrected ratio of at least 4).

As commonly accepted for routine Luminex based HLA antibody testing, all results were individually reviewed for plausibility.

### 2.3 Statistics

For descriptive statistics, differences between groups were described using the Pearson chi‐squared test and Mood median test, where appropriate. Death‐censored graft survival was evaluated with Kaplan–Meier‐curves and significant differences determined using log‐rank analyses. A *p*‐value below 0.05 was considered significant. Calculations were performed by SPSS (IBM Corporation, Armonk, NY, USA).

## 3 Results

The characteristics of the 49 DSA‐positive cases and 1216 controls are shown in Table 1. DSA‐positive patients were more likely to be female and had higher PRA‐values compared to the controls. Some patients had PRA‐values of 0%, because these data were drawn from the Eurotransplant database and mostly based on CDC‐reactivity. As expected, the proportion of re‐transplant patients was higher in the DSA‐positive group. There was no difference between the two groups with respect to AB0 incompatibilities and all patients were transplanted with a negative lymphocytotoxic crossmatch.

**TABLE 1:** Characteristics of included patients and controls.

		LD without DSA	LD with DSA^a^
*N*		1216	49
Age		46 (33–55)	47 (37–53)
Rec. sex (F)		438 (36%)	28 (57%)
Current PRA%		0 (0–0)	0 (0–26)
Highest PRA%		0 (0–0)	15 (0–82)
DSA HLA class	No DSA	n.a.	0 (0%)
	Cl. I	n.a.	22 (45%)
	Cl. II	n.a.	13 (27%)
	Cl. I + II	n.a.	14 (29%)
Preemtive Tx		387 (32%)	9 (18%)
Dialysis pre Tx	Years	0 (0–1)	1 (0–2)
Don. age		53 (47–60)	53 (46–57)
Don. sex F		736 (61%)	25 (51%)
Cold ischemia time		151 (120–180)	150 (120–190)
MM HLA ABDR		3 (2–4)	4 (3–5)
Number of previous Tx	0	1152 (95%)	35 (71%)
	1	56 (5%)	12 (24%)
	2	8 (1%)	2 (4%)
Induction therapy^b^	ATG	62 (5%)	11 (23%)
	ATG/CD20	6 (0.5%)	2 (4%)
	ATG/IL‐2	1 (0.1%)	0 (0%)
	ATG/IL‐2/CD20	2 (0.1%)	0 (0%)
	CD20	15 (1.2%)	2 (4%)
	CD20/Alemtuzumab	3 (0.2%)	0 (0%)
	IL‐2	670 (55%)	19 (39%)
	IL‐2/CD20	119 (10%)	9 (18%)
	None	177 (17%)	6 (12%)
Initial immunosuppression^b^	Tac/MMF/steroids		42 (86%)
	CsA/MMF/steroids		2 (4%)
	Other		5 (10%)
Desensitisation	No desensitisation	914 (75%)	32 (65%)
	HLA ab desensitisation	34 (3%)	6 (12%)
	ABO‐incompatible Tx	266 (22%)	11 (22%)

*Note:* Categorical values are given as number (%), numerical values as median (interquartile range).

Abbreviations: DSA, donor‐specific HLA antibodies; PRA, panel‐reactive‐antibodies.

^a^ LD with DSA, tested by all three providers.

^b^ Incomplete data from control patients.

**FIGURE 1:**
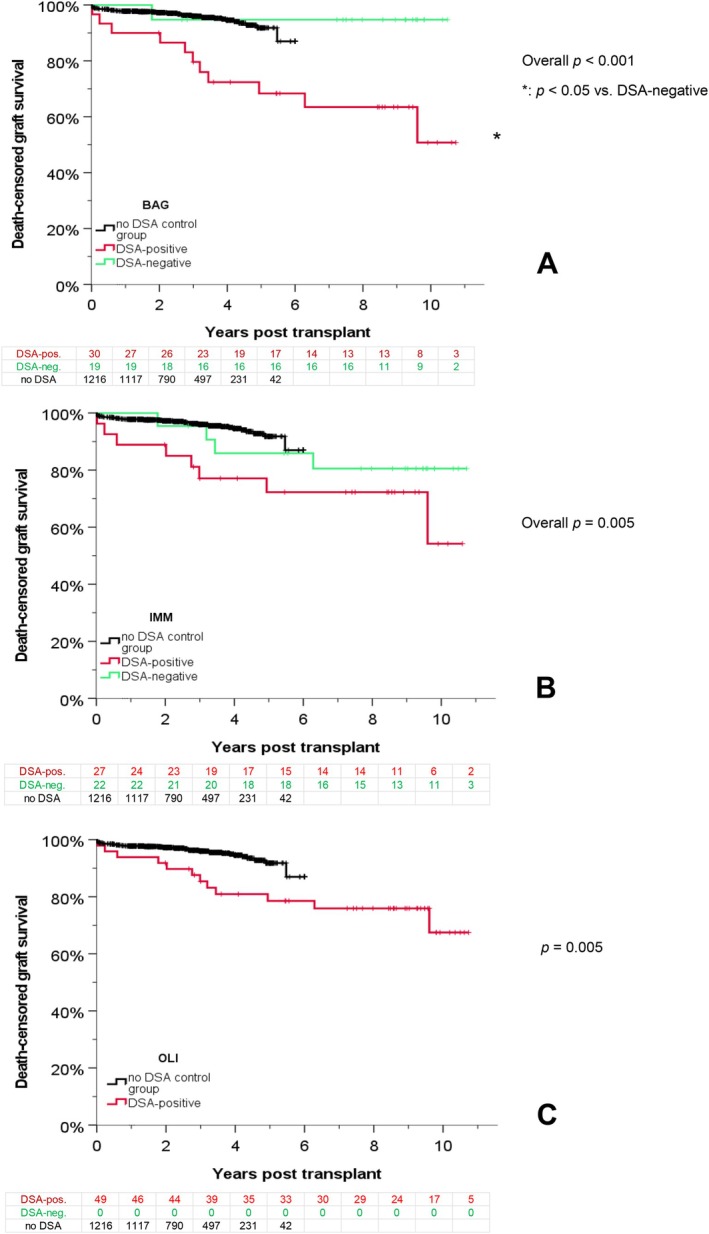
Graft survival and positivity for HLA DSA in three different single antigen assays. The graft survival rate in patients with DSA detected by the assays BAG (A), IMM (B) and OLI (C) is shown compared to patients tested DSA‐negative by these assays, and to patients without any DSA from the original study. For OLI, no ‘DSA‐negative’ patients were observed, because all DSA detectable by BAG and/or IMM were also detectable by OLI.

The 10‐year graft survival rate in patients with HLA DSA is shown in Figure 1, split up according the results of all three assays (BAG, IMM and OLI). OLI was the most sensitive assay and classified all sera as DSA‐positive, that were DSA‐positive by BAG and/or IMM.

**FIGURE 2:**
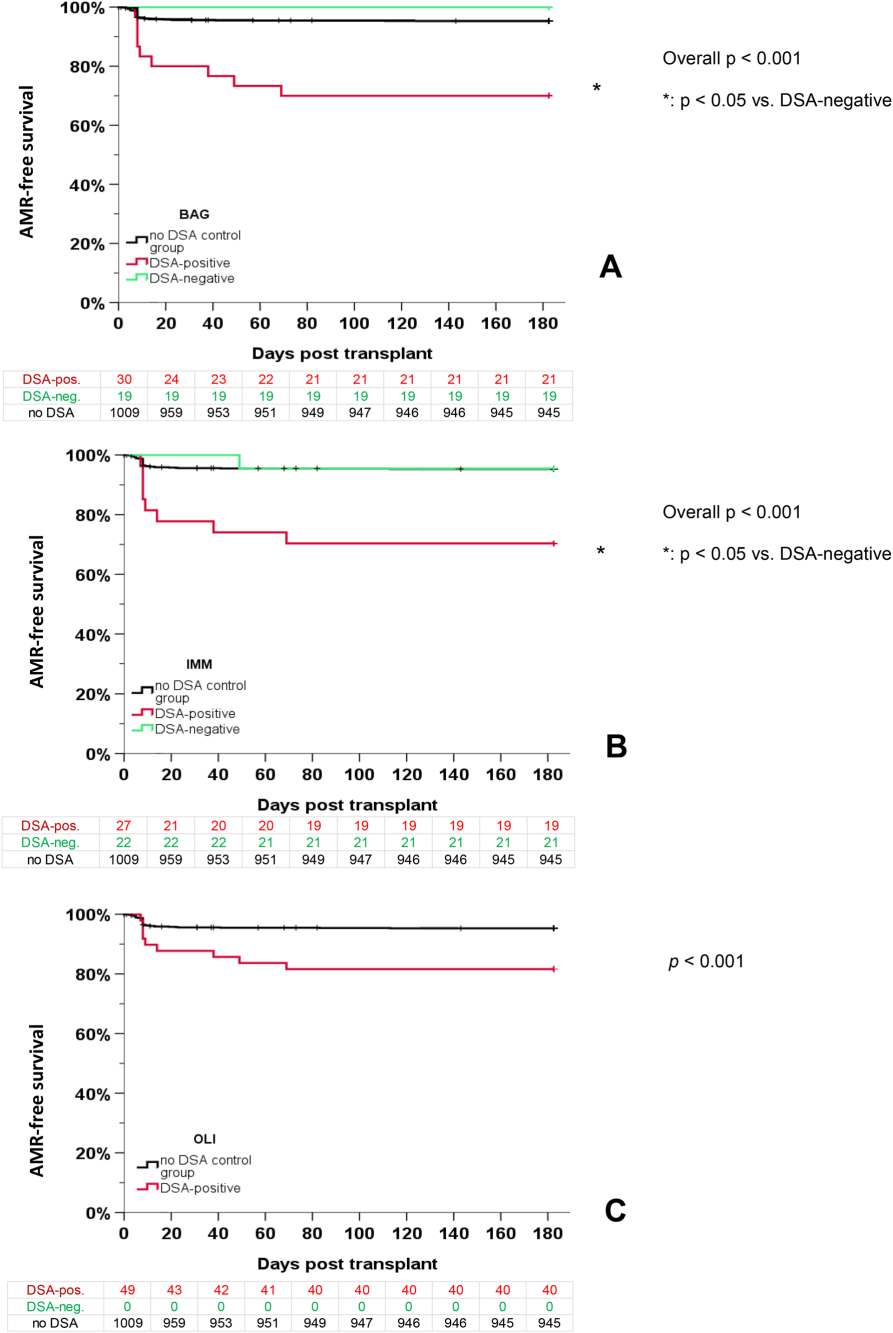
Rejection free survival and HLA DSA positivity in three different assays. The AMR‐free survival rate in patients with DSA detected by the assays BAG (A), IMM (B) and OLI (C) is shown compared to patients tested DSA‐negative by these assays, and to patients without any DSA from the original study. For OLI, no ‘DSA‐negative’ patients were observed, because all DSA detectable by BAG and/or IMM were also detectable by OLI.

The AMR free survival rate stratified for the HLA DSA positivity for all three assays is shown in Figure 2. Our analyses clearly show that DSA‐positive patients had worse outcome than DSA‐negative patients, independent of the assay used.

**FIGURE 3:**
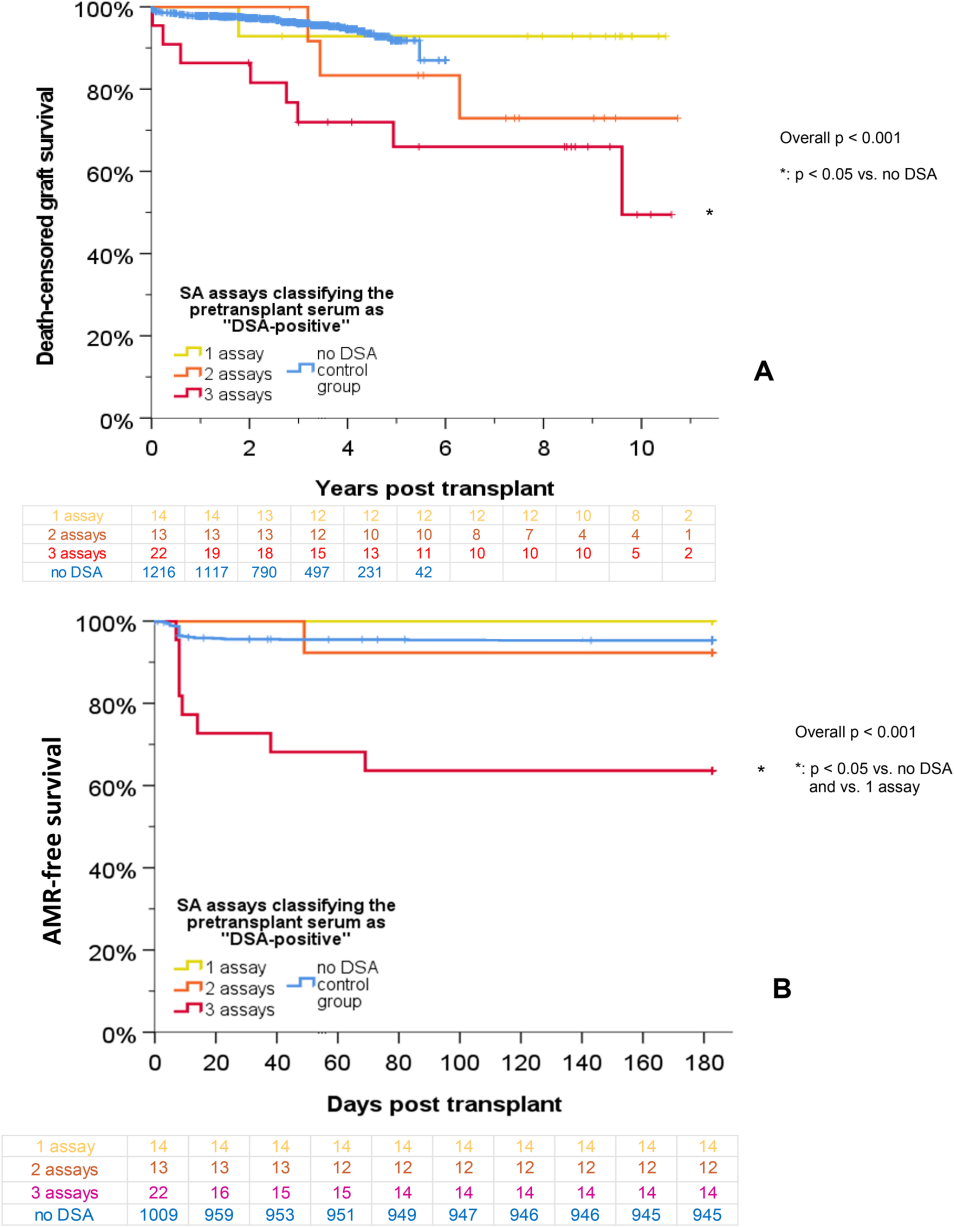
Outcome according to number of assays classifying serum samples as ‘DSA‐positive’. The graft survival (A) and the AMR‐free survival (B) rates in all patients are shown with respect to the number of SA assays (BAG, IMM and OLI) classifying the serum samples as HLA ‘DSA‐positive’.

Figure 3 illustrates the graft survival and the AMR free survival rates in all patients divided by the number of SA assays (BAG, IMM and OLI) classifying the serum samples as DSA‐positive. Fourteen sera were DSA‐positive in only one assay (always OLI), 13 DSA‐positive in two assays (eight sera for BAG and OLI, five sera for IMM and OLI) and 22 sera were DSA‐positive in all three assays. The AMR rate was increased in patients showing DSA in all three SA assays, both when compared to the controls and when compared to DSA‐positive patients in only one assay (*p* < 0.05).

**FIGURE 4:**
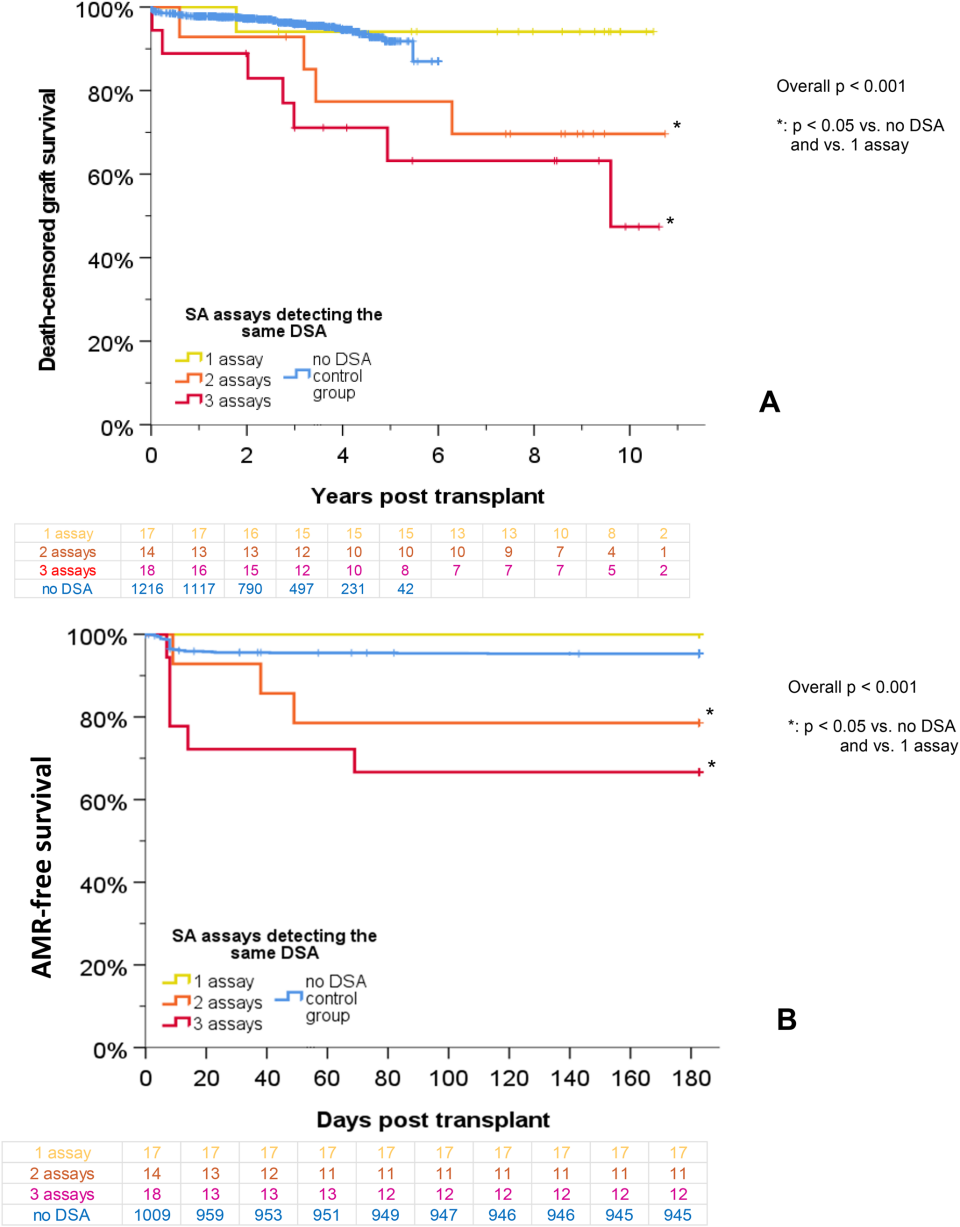
Outcome according to the number of assays detecting at least one identical DSA. The graft survival (A) and the AMR‐free survival (B) rates in all patients are shown with respect to the number of SA assays (BAG, IMM and OLI) detecting one (yellow line), two (orange line) or three (red line) identical HLA DSA.

The outcome according to the number of SA assays detecting at least one identical HLA DSA specificity is shown in Figure 4. In 18 sera, at least one identical DSA was detected by all three assays. The corresponding patients had significantly higher AMR rates and reduced 10‐year graft survival rates. In 14 sera, the same DSA was detected by IMM and OLI, or by BAG and OLI, respectively. These patients had lower AMR free survival rates, but no significant difference in the 10‐year graft survival. In 17 sera, DSA were detectable by one or more SA assays, but no specificity was positive in more than one assay (in all sera DSA were detected by OLI, in one additionally another DSA by IMM and in two another DSA by BAG). In these patients, AMR free survival and 10‐year graft survival were similar to DSA negative patients.

**FIGURE 5:**
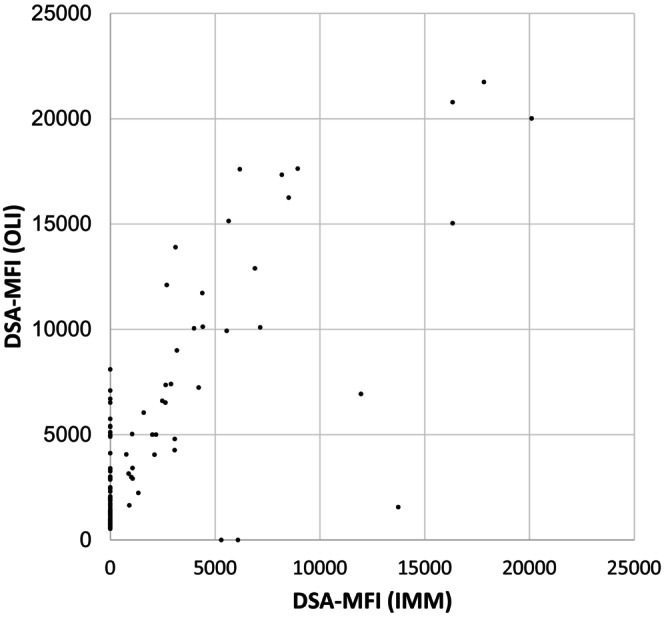
Comparison of the reaction strength of the bead‐based SA assays for all 106 DSA found in the 49 sera.

Next, Figure 5 shows a MFI comparison of the bead‐based SA tests from OLI and IMM. MFI values for IMM were generally weaker than for OLI. Strong DSA of 9000 MFI or more were always detected by both methods, while two weaker DSA were detected by IMM but not by OLI and 65 by OLI but not by IMM. Of the 65 DSA detected by OLI but not by IMM, 49 DSA had values below 3000 MFI, 8 DSA had between 3000 and 4999 MFI and 8 DSA had MFI values of 5000 or more. The strongest DSA detected by OLI but not by IMM had 8098 MFI, the strongest DSA detected by IMM but not OLI had 6094 MFI.

**FIGURE 6:**
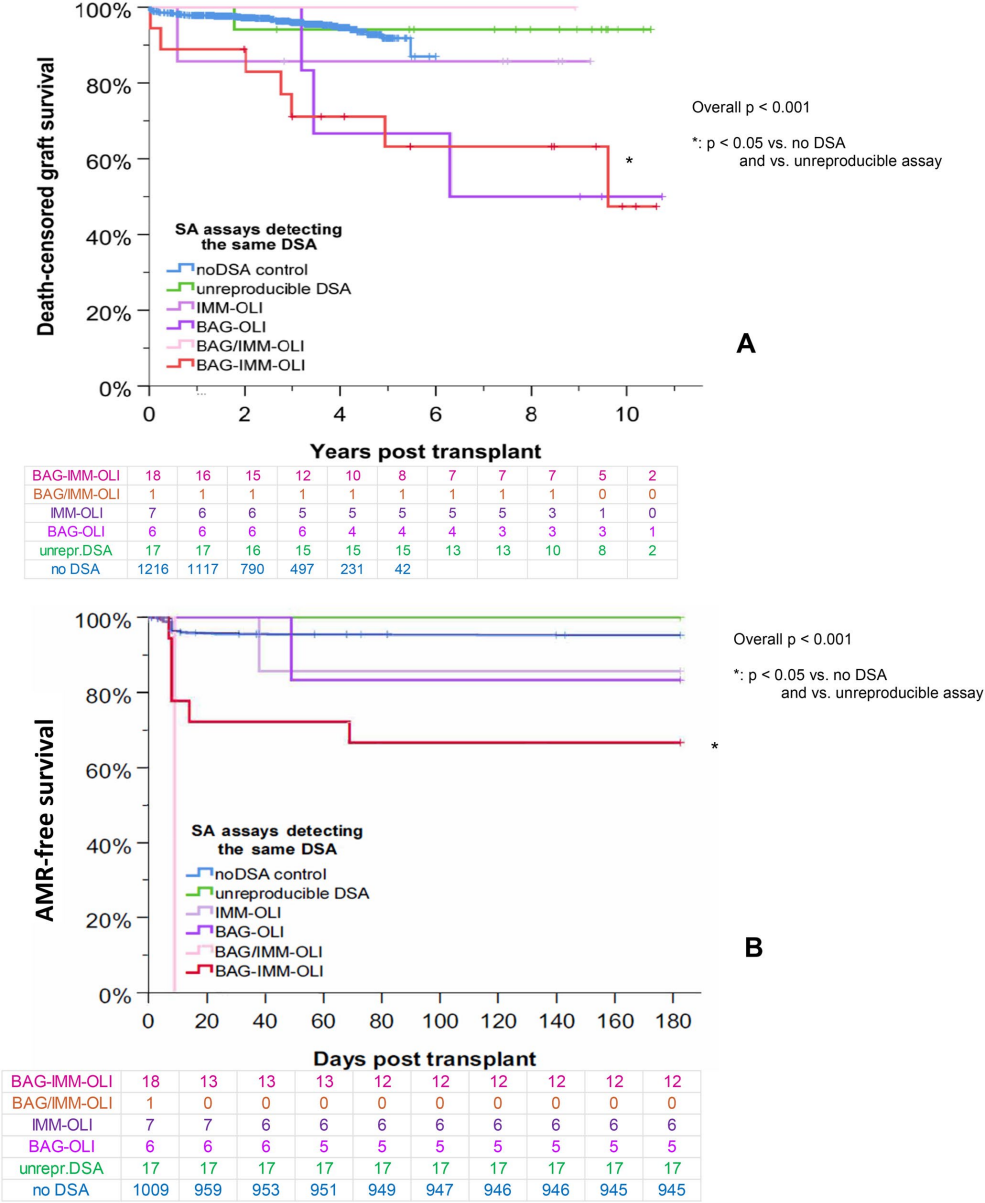
Outcome according to the different assays detecting identical DSA. The graft survival (A) and the AMR‐free survival (B) rates in all patients are shown with respect to the different SA assays (BAG, IMM and OLI) detecting the same DSA and compared to sera that are only DSA‐positive in one assay (unreproducible) and HLA DSA negative controls.

Finally, we stratified according to the combination of assays detecting the same DSA specificity, which did not reveal additional significant information (Figure 6). There was only a significant association with worse AMR free survival and graft survival when all three assays showed the same specificity compared with the no‐DSA controls or the specificities shown in only one assay (which was defined as ‘unreproducible DSA’).

Also, evaluation of overall graft survival showed similar results as reported for death‐censored graft (data not shown).

## 4 Discussion

This work is unique in comparing the prognostic value of HLA DSA detected by the recently released HISTO SPOT microspot array (BAG) together with the established Luminex bead based arrays (OLI and IMM) in the context of kidney transplantation outcome. We could show that all three SA assays comprising comparable sets of antigens were suitable for the reliable detection of strong reacting HLA antibodies in kidney transplant patients. OLI was shown to be the most sensitive assay, but also prone to positive results not detected by any of the other assays. DSA detected by only one assay were neither associated with decreased graft survival, nor with increased incidence of AMR. While such reactions were rare for the BAG and IMM assays, the latter assays missed some DSA associated with an increased risk for AMR. There was no clear difference in the prognostic value of DSA detected by BAG and OLI versus DSA detected by IMM and OLI, which supports the finding that IMM and BAG have a generally lower sensitivity when compared to OLI.

Comparative analyses for all three methods have been rarely reported so far und are completely lacking in the context of organ transplantation outcome. However, a recent study comprising patients with platelet refractoriness could also confirm differences in sensitivity between OLI and BAG: Although the BAG assay is less sensitive than OLI, this assay could be used for the selection of HLA‐compatible platelet products [17]. The authors also evaluated the clinical impact by checking the efficacy of platelet concentrates by the corrected count increment at 24 h as a function of the HLA specificities detected by the different SA tests. Interestingly, this study also revealed that the BAG method is less sensitive than the standard OLI but is relatively similar to the OLI assay in combination with the supplementary C1q kit. Supplementary C1q or C3d assays are available for OLI and IMM, but they were not included in our study described here.

The high sensitivity of the Luminex bead based SA assay is widely acknowledged nowadays and was again confirmed by our study showing the capability to detect most relevant HLA antibodies. On the other hand, the occurrence of unspecific or ‘natural’ antibodies by direct SA bead testing with OLI and IMM may lead to an inaccurate assignment of potentially clinically relevant specificities. So, most laboratories rely on a step‐wise HLA antibody screening strategy starting with a screening assay with lower sensitivity first before proceeding the sample to the sensitive and expensive SA specification assay, although the weaknesses in sensitivity of the screening assays are known [18, 19, 20, 21].

Examining peri‐biopsy serum samples from kidney transplant recipients with possible AMR, Burballa et al. [8] could show that performing SA bead testing only in case of positive initial Luminex based screening failed to identify clinically relevant HLA antibodies. To avoid this limitation in sensitivity, the authors recommend direct SA bead testing for post‐transplantation monitoring in case of possible AMR. On the other hand, direct SA bead testing for general post‐transplantation monitoring or even for pre‐transplant diagnostics would create high additional costs for the laboratories and poses the risk of defining clinically irrelevant unacceptable antigens for waiting list patients. Regardless of the indication for SA bead testing, a detailed HLA antibody pattern analysis considering denatured epitopes in DSA reporting combined with a thorough interpretation of immunising events is recommended. If applied accurately, such a procedure has been shown to increase the quality of DSA testing [3]. For our study, we did not systematically consider antibodies against denatured or ‘natural’ antigens. However, we retrospectively performed a separate analysis for the OLI assay stratifying for antibodies against possibly denatured antigens, which did not reveal significant differences compared to the results showed above, probably due to the low number of cases. Although positive reactions due to denatured HLA antigens have also been reported in IMM and BAG assays by Pandrey and Harville [22], there is only scarce information available on affected HLA alleles or the frequencies of putative false positive reactions, which therefore prevented comprehensive analyses in the present study.

An exact definition of HLA antibodies is mandatory for special organ allocation programmes for highly immunised transplant patients, for example, the Eurotransplant Acceptable Mismatch (AM) program. The eligibility of the patients for the AM program and the definition of acceptable HLA mismatches is currently validated by the Eurotransplant Reference Laboratory (ETRL) for every single patient case. Thus, the ETRL has recently made extensive efforts to elucidate the impact of MFI values and the differences in the SA bead assay performance by the two vendors OLI and IMM [12, 23]. Findings of our study may provide substantial help for the assessment of the HLA antibody screening results the laboratories report to the ETRL seeking for inclusion of their patients in the AM program. It should be discussed, whether parallel testing using SA assays from different providers with different sensitivities might be helpful for the separation of strong DSA from weaker ones. In general, the increased immunological risk due to the presence of DSA has always to be carefully weighed against the risk due to increased waiting time for each individual patient.

There are limitations of our retrospective study, which comprises only a low number of selected patients. For sure, larger studies are necessary to confirm the result reported here and to perform more detailed analyses of differences between the assays regarding sensitivity and specificity. Next, the diagnosis of antibody‐mediated rejections is another weakness in our study cohort. Most centres did not perform protocol biopsies, so most AMR cases were diagnosed by indication biopsies and subclinical rejections might have been missed. We aimed to get as accurate data as possible by summarising not only biopsy‐proven antibody‐mediated rejection but also presumed antibody‐mediated rejection in the absence of biopsies. A further potential limitation of this study is that both, details of the procedures used for antibody testing prior to transplantation, as well as consequences according to the test results with respect to the transplant decision, varied widely between the different transplant centres. Also, the strategies for long‐term immunosuppression were heterogeneous, and no detailed data about modifications of the initial immunosuppressive therapy during the observation period were available. However, on the other hand this heterogeneity is also an important strength of this work, which shows a clear prognostic value for kidney transplant outcome in a real‐world‐scenario.

Our results point out that all three SA assays compared here are able to reliably detect strong DSA. OLI was shown to be the most sensitive assay, but also prone to positive results not associated with impaired outcomes. Antibodies detectable with only one SA assay were not associated with impaired outcome and should therefore not be considered for graft selection. Future studies are needed to determine how many OLI‐only reactions are caused by very weak DSA and how many by reactions with denatured beads. While such reactions were rare for BAG and IMM assays, the latter assays missed some DSA associated with an increased risk for AMR.

So, while all three SA assays seem suitable for routine testing, comprehensive SA testing combining different methods might be helpful for optimal risk stratification in selected cases (e. g. broadly immunised patients with many reactions of medium intensity).

An overview about the different study groups included in this work is available as Supporting Information (Figure S1).

## Author Contributions

F.M.H., T.K., M.H., M.K., M.L., M.V.: Investigation, methodology, patient sample and data acquisition, data curation, writing. K.A., K.B., G.E., U.E., A.E., T.F., F.G., M.G., C.H.‐L., C.H., S.K., N.L., C.M., M.N., L.R., S.S., G.S., J.S., V.S., F.S., B.M.S., C.S., M.V., D.Z.: patient sample and data acquisition. M.Z.: conceptualisation, project administration, funding acquisition, investigation, methodology, patient sample and data acquisition, data curation, writing.

## Funding

The study was supported by a grant of the Stiftung Transfusionsmedizin as well as by free reagents provided by the companies Immucor/Werfen, One Lambda/ThermoFisher and BAG Health Care.

## Conflicts of Interest

The authors declare no conflicts of interest.

## Supporting information


**Figure S1:** Clarifying overview about the different study groups.

## Data Availability

The data that support the findings of this study are available on request from the corresponding author. The data are not publicly available due to privacy or ethical restrictions.
