# Supplementary material for: Comparison of the Prognostic Value of Three Different Single HLA Based Antibody Detection Assays
Source: HLA. 2026 Apr 25;107:e70734. doi: 10.1111/tan.70734 (PMC13109763; doi:10.1111/tan.70734)
Supplement: Supplementary file 1 — Figure S1: Clarifying overview about the different study groups. [file TAN-107-e70734-s001.docx]

**Figure S1: Clarifying overview about the different study groups**

The following supplemental figure provides a clarifying overview of the study groups.

We evaluated separately

- the number of SA tests classifying the serum as DSA-positive irrespective, whether the same or different DSA-specificities were detected (Part 1), and
- the number of SA tests detecting at least one identical DSA specificity (Part 2).

**Part 1 Part 2**

22 DSA-positive by three assays (irrespective whether same or different DSA)

18 sera with at least one identical DSA detected by three assays

**Study cohort**:

49 pretransplant sera from 49 patients with living kidney donation
(one serum per patient)

13 DSA-positive by two assays (irrespective whether same or different DSA)

14 sera with at least one identical DSA detected by two assays

14 DSA-positive by one assay

17 sera in which no DSA-specificity could be detected by more than one assay
